# Holt-Oram syndrome: a rare clinical image

**DOI:** 10.11604/pamj.2023.45.25.38864

**Published:** 2023-05-08

**Authors:** Kshitij Aviraj Singh, Amar Taksande

**Affiliations:** 1 Department of Paediatrics, Jawaharlal Nehru Medical College, Dutta Meghe Institute of Higher Education and Research, Sawangi Meghe, Wardha, Maharashtra State, India

**Keywords:** Skeletal manifestations, congenital heart defect, radial bone defect, ventricular septal defect, Holt-Oram syndrome

## Image in medicine

Holt-Oram syndrome is a rare autosomal dominant disorder presenting skeletal abnormalities of the upper limbs (hands and arms) with an underlying structural and/or conduction heart defect. The diagnosis is often made on clinical presentation. An associated cardiac defect may consist of complex congenital heart defects, conduction defects, and arrhythmias. Patients with Holt-Oram syndrome have at least one skeletal deformity in the upper limb, which may include an abnormal or missing wrist bone on an X-ray. The skeletal deformity may vary in severity and presentation, and include a thumb that looks like a finger, a thumb missing on hand, unequal length or underdeveloped upper arm bones, a partial or complete absence of bones in the forearm, and collar bone or shoulder blade abnormalities. Here, we are reporting a case of Holt-Oram syndrome in a five-year male child with ventricular septal defect (VSD), unusual skeletal deformity of the hypoplastic humerus, along with radial and ulnar bone defect, and a unique feature of Holt-Oram syndrome seen in our case

**Figure 1:**
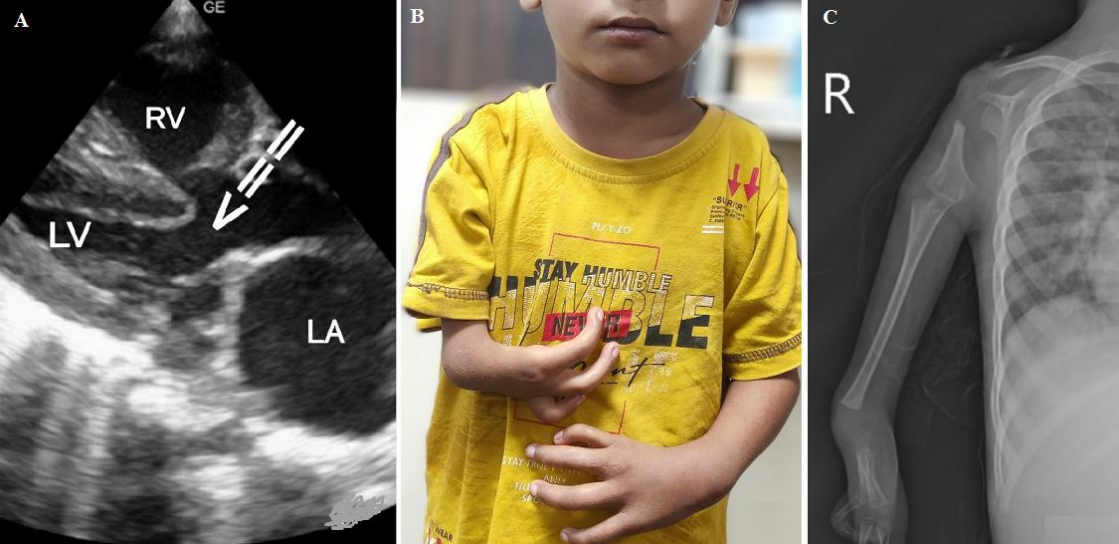
A) 2-D echo of the patient showing ventricular septal defect (VSD) (LA: left atrium; RV: right ventricle; LV: left ventricle); B) clinical photo of the patient with upper limb skeletal deformity; C) X-ray of right upper limb showing hypoplastic humerus with radial and ulnar bone defect

